# Initial Steps for the Development of a Phage-Mediated Gene Replacement Therapy Using CRISPR-Cas9 Technology

**DOI:** 10.3390/jcm9051498

**Published:** 2020-05-16

**Authors:** Jordi Yang Zhou, Keittisak Suwan, Amin Hajitou

**Affiliations:** Phage Therapy Group, Department of Brain Sciences, Imperial College London, London W12 0NN, UK; jordi.yang-zhou18@imperial.ac.uk

**Keywords:** bacteriophage, gene therapy, CRISPR-Cas9, p53, lung cancer, tumour suppressor gene replacement therapy

## Abstract

p53 gene (*TP53*) replacement therapy has shown promising results in cancer gene therapy. However, it has been hampered, mostly because of the gene delivery vector of choice. CRISPR-Cas9 technology (clustered regularly interspaced short palindromic repeats/CRISPR-associated protein 9) can knock out the mutated *TP53* (*mutTP53*), but due to its large size, many viral vectors are not suitable or require implemented strategies that lower the therapeutic efficiency. Here, we introduced a bacteriophage or phage-based vector with the ability to target cancer cells and aimed to investigate the feasibility of using this vector to deliver CRISPR-Cas9 transgene in human lung adenocarcinoma cells. First, we produced a tumour-targeted bacteriophage carrying a CRISPR-Cas9 transgene cassette. Next, we investigated any negative impact on vector titers via quantitative polymerase chain reaction (qPCR) and colony-forming agar plate. Last, we combined Western blot analysis and immunofluorescence staining to prove cell transduction in vitro. We showed that the tumour-targeted bacteriophage can package a large-size vector genome, ~10 kb, containing the CRISPR-Cas9 sequence without any negative impact on the active or total number of bacteriophage particles. Then, we detected expression of the Cas9 in human lung adenocarcinoma cells in a targeted and efficient manner. Finally, we proved loss of p53 protein expression when a p53 gRNA was incorporated into the CRISPR-Cas9 phage DNA construct. These proof-of-concept findings support the use of engineered bacteriophage for *TP53* replacement therapy in lung cancer.

## 1. Introduction

Lung adenocarcinoma is one of the most frequent causes of cancer-related deaths worldwide. Based on data from the World Health Organization (WHO), in 2018 there were approximately 2.09 million cases and 1.76 million deaths. Unfortunately, current treatments have shown few benefits and are the main reason for lung cancer-related deaths due to their aggressive nature.

Gene therapy has emerged as a promising tool to tackle a wide range of diseases, including cancer. Identifying tumour-suppressor genes has been pivotal for the development of anticancer therapies. In particular, the tumour suppressor p53 gene (*TP53*) has been regarded with interest by many gene therapists since its discovery in 1979 [1]. High throughput screenings in lung cancer patients show that half of tumours present mutations in the *TP53 (mutTP53)* [2,3,4]. The concept of “*TP53* gene replacement therapy” consists of restoring the *wild-type* (*wt*) *TP53* gene as a means to suppress tumour growth and progression by delivery and expression of a *wt TP53* gene in tumours. Reported studies have shown promising results in clinical trials by using a replication-deficient p53-expressing adenoviral vector (Ad-p53 vector). The treatment proved to have an effective anti-tumour effect with low toxicity to normal tissues, and enhanced tumour sensitivity to conventional chemotherapy and radiotherapy [5,6]. However, recently, studies have reported that after introducing the *wt TP53* gene copy, some stable aberrant forms of p53 can interfere with the therapy [7,8,9]. The p53 tetramerizes before reaching the nucleus. In the presence of aberrant p53, the former is assembled in atetramer impeding the complex to interact with its approximately 500 target genes. Using the CRISPR-Cas9 technology can overcome this problem by removing the aberrant form of *TP53* before introducing the healthy *wt TP53* copy. Yet, the nature of mammalian vectors brings serious limitations such as low transduction rates or immunogenicity, which is an obstacle to repeated vector delivery, therefore highlighting the need for a more suitable delivery vector. In addition, the broad tropism of mammalian viruses for healthy tissues limits their efficiency in systemic delivery due to general off-target effects [10]. We propose to use a bacteriophage-based viral vector to overcome these limitations. Our vector is a genetically engineered M13 filamentous bacteriophage, or phage, that delivers transgene expression cassettes flanked by the Inverted Terminal Repeat (ITR) elements of the human adeno-associated virus 2 (AAV2) [11]. The phage pIII minor coat protein displays the double cyclic RGD4C (CDCRGDCFC) ligand, which binds to the tumour-specific α_v_β_3_ and α_v_β_5_ integrin receptors [11]. The vector was reported to be internalized via a dynamin and clathrin dependent system [12]. Previously, we noticed that 100 percent of the vector was internalized into cells after transduction [12]. However, the endosomal-lysosomal degradative pathway is redeemed as a major intracellular limitation to RGD4C phage vectors, since the particles are sequestered and degraded within the lysosomes [12]. This leads to expression of the transgene in up to 15% of the transduced cells. We recently developed a bacteriophage-based vector bearing large endosomal escape peptides (EEP) from animal viruses on the recombinant pVIII (r-pVIII), since the wild-type pVIII major coat protein of phage can only display short peptides [13]. Thus, we further constructed an RGD4C phage by displaying the histidine-rich H5WYG EEP on the r-pVIII coat proteins. The latter phage-based vector boosted vector escape from the endo-lysosome pathway, and subsequently improved gene delivery tin tumour cells in vitro and in mouse models of solid tumours in vivo, following intravenous administration [13]. After escaping from the endosomes, the ITR-flanked transgene cassette accumulates in the nucleus, resulting in gene expression [14].

Bacteriophage-based vectors have some potential advantages over mammalian viral vectors since (i) bacteriophages are not natural pathogens of mammals and have no native tropism for mammalian cells and tissues, allowing their delivery through the systemic route [15]; (ii) genetically engineered bacteriophages acquire tropism for solid tumours, resulting in successful targeted systemic administration [11,13,16,17,18,19,20,21,22,23]; (iii) repeated vector dosing is safe and does not show a negative impact on therapy efficacy [11,18,23]; (iv) phage vectors can accommodate large size transgenes cassettes over existing vectors with minimal effects on the vector titer; (v) the vector is stable and viable at 4 °C for long periods [24], whereas other mammalian viruses require ultralow temperatures for storing and transporting; and finally (vi) manufacturing of genetically engineered bacteriophages is simple and cost-effective, yet efficient.

In this study, we explored the feasibility of using our M13-based RGD4C bacteriophage vector, with endosomal escape ability via display of the H5WYG EEP on the recombinant r-pVIII major coat proteins, for targeted and efficient delivery of CRISPR-Cas9 to human lung cancer cells. The outcomes of this project could bring a simple, cost-effective and safe treatment for lung cancer.

## 2. Materials and Methods

### 2.1. Cell Culture

Human embryonic kidney HEK293 cells were purchased from the American Type Culture Collection (ATCC^®^ CRL-1573™) and the A549 human lung adenocarcinoma cells were a gift from Professor Ian M. Adcock (Imperial College London, London, UK). All cell lines were maintained in Dulbecco’s modified Eagle’s medium (DMEM) supplemented with 10% foetal bovine serum (FBS), penicillin (100 units/mL, Sigma-Aldrich, Haverhill, UK), streptomycin (100μg/mL, Sigma-Aldrich, Haverhill, UK) and 1% GlutaMAX (ThermoFisher Scientific, Hemel Hempstead, UK).

### 2.2. Cas9 Phage Plasmid Construction

**Figure 1:**
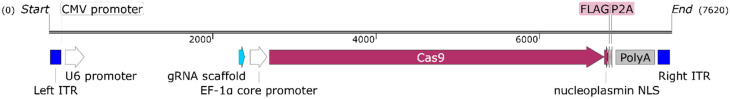
Map of the CRISPR-Cas9 (clustered regularly interspaced short palindromic repeats/CRISPR-associated protein 9) transgene cassette, 7620 bp, inserted within the M13 phage genome. Left and right ITRs (Inverted Terminal Repeats), from AAV2, flank the CRISPR-Cas9 cassette. The guide-RNA (gRNA) scaffold incorporates a target site to make CRISPR-Cas9 protein functional; there is a ~1.8 kb space between U6 promoter and the gRNA scaffold. Image generated by SnapGene (GSL Biotech, Chicago, IL, USA).

Cas9 expression cassette (Figure 1) was modified from the lentiCRISPRv2 (Addgene, Watertown, USA) using the following forward (Fw) and reverse (Rev) primers: Cas9-Fw (5′-*CGCTAACGCGTGGACAGCAGAGATCCAGTTTGGT*) and Cas9-Rev (5′-*CGCTAGTCGACAGAAGTTTGTTGCGCCGGA*). These primers contain restriction enzyme recognition sites Mlu-I and Sal-I, at the 5′ end, respectively. The pAAV-GFP control plasmid (Cell Biolabs Inc., Slough, UK) and the PCR amplicon fragment were ligated at a molar ratio 1:3 (plasmid:fragment) after digestion with MluI and SalI restriction enzymes. Next, the resulting final phage plasmid was subjected to DNA sequencing analysis, (Eurofins, Constance, Germany), using a Cas9 sequencing primer (5′-*TTGAGATCCTTTTTTTCTGCGCGTAA*). In order to incorporate the p53 gRNA (5′-*AGCACATGACGGAGGTTGTG*), we ligated the fragment between the U6 promoter and the gRNA scaffold shown in Figure 1. The gRNA targets exon 5 of the *TP53 gene*.

### 2.3. M13 (Non-Targeted) and RGD4C (Targeted) Bacteriophage-Based Vector Production and Titration

Vectors were generated following a phage-based vector production, as we reported [11,13,20]. The phage capsid contained an additional recombinant r-pVIII major coat protein for the display of H5WYG (GLFHAIAHFIHGGWHGLIHGWYG) histidylated fusogenic peptide with endosomal escape capacity, derived from the N-terminal of the HA2 subunit of the human influenza virus hemagglutinin [13]. Viral particles were produced in *Escherichia coli* TG1 strain and sterile-filtered through a 0.45µM filter [11]. The total number of particles was measured by traditional qPCR (quantitative Polymerase Chain Reaction). Analysis was performed using the CFX96 Touch Real-Time PCR Detection System. PCR reactions were performed in 15 µL of final volume using the PowerUp™ SYBR™ Green Master Mix (Thermo Fisher Scientific, UK), supplemented with 100µM sense primer targeting AAV2 ITR (5′-*GGAACCCCTAGTGATGGAGTT*) and 100µM antisense primer targeting AAV2 ITR (5′-*CGGCCTCAGTGAGCGA*) [25]. A standard curve was prepared using known concentrations of ITR-containing plasmid, and samples were diluted 1:5000 in sterile water. The PCR cycling parameters contained an initial denaturation at 98 °C for 3 min, then 98 °C for 15 s and annealing or extension at 58 °C for 30 s. The plate was read and step 3 was repeated 39 times, followed by a melt curve stage. Data analysis was performed using the Bio-Rad CFX Manager v3.1 and expressed as genome-containing particles/µL. Active particles were titrated based on an agar plate counting system [11,17] and expressed as bacterial transducing units (TU/µL).

### 2.4. Transfection of the Cas9 Phage Plasmid with Polyethyleneimine (PEI)

Cells were grown in Serum Free Media (SFM) for 1 h prior to transfection. A mix of 1:3 ratio (µg DNA: µg PEI (QBiogene, Carlsbad, CA, USA)) was prepared in SFM, vortexed and incubated for 10 min at room temperature (RT). Then the transfection mix was added dropwise into each well of cells. After 3 h post-transfection, the transfection media was replaced with complete DMEM.

### 2.5. Cell Transduction by Phage Vectors

The procedure is based on our previous protocol for cell transduction by phage vectors [17]. Briefly, HEK293 and A549 cells were plated at 5 × 10^5^ cells/well in a poly-D-lysine coated 12-well plate. Phage viral stock was diluted in serum-free medium (SFM) to achieve the desired Transducing Unit per cell ratio (TU/cell). The mix was incubated at RT for 15 min. Cells were transduced by drop wising the mix and overnight (O/N) incubation. The next day, the transducing medium was replaced with 500µL complete DMEM. Untreated cells were used as a control. The culture continued for 6 days before carrying out immunocytochemistry.

### 2.6. Western Blot

Cell lysates were prepared using 100 µL 1× SDS sample buffer, followed by sonication for 20 s. Samples were heated at 95 °C for 5 min, cooled on ice and centrifuged for 5 min before loading to SDS-PAGE and transferred to a nitrocellulose membrane. The membrane was blocked with 5% skimmed milk for 1 h. The primary antibodies used were mouse anti-Cas9 (1:1000; Cell Signalling Tech, London, UK) and rabbit anti-GAPDH (1:5000; Santa Cruz Biotechnology, Texas, USA).

### 2.7. Immunofluorescence Staining

Cells were fixed in 4% paraformaldehyde (Merck, Darmstadt, Germany) for 15 min at RT, and permeabilized for 15 min with 0.01% Triton x-100. Cells were then incubated with 2% filtered BSA (bovine serum albumin) for 1 h before primary antibody incubation (mouse anti-Cas9 antibody and/or rabbit anti-p53 antibody, 1:800 in 1% BSA), O/N, 4 °C. After 3 washes with phosphate buffer saline (PBS), cells were labelled either with Alexa Fluor^®^ 594 goat anti-mouse or anti-rabbit Igs, and/or Alexa Fluor^®^ 488 goat anti-mouse Ig (1:500; Life Technologies, Darmstadt, Germany), and Alexa Fluor^®^ 488 Phalloidin (1:300; Life Technology, Germany) in 6-diamidino-2-phenylindole (DAPI) (Sigma; 1:4000 dilution in PBS). Cells were incubated 1 hour at RT in the dark. After 3 washes with 1% BSA, 3 washes with PBS and 1 wash with water, coverslips were mounted. Controls were treated with the secondary antibody only. Cells were imaged with a DMi8 advanced confocal fluorescence microscope (Leica Microsystems, Wetzlar, Germany). Images were analysed using LAS× software (Leica Microsystems, Germany).

### 2.8. Statistical Analysis

To evaluate any potential alteration in viral production due to CRISPR-Cas9 genome vector size, we compared the total and active particle of the former vector and a control. The experiment was repeated 3 times (n = 3) independently, and each replicate was measured 3 times. Statistical analysis was subjected to paired Student’s *t* test with significance at * *p* < 0.05.

## 3. Results

### 3.1. Positive Validation of the CRISPR-Cas9 Phage Plasmid

**Figure 2:**
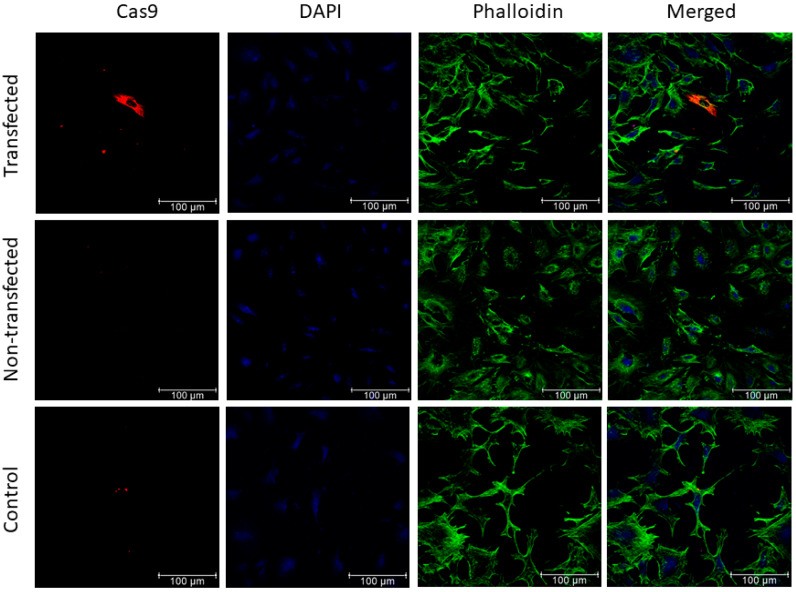
A549 cells transfected with CRISPR-Cas9 phage plasmid. Cells were stained with anti-Cas9 antibody (red), phalloidin-Alexafluoro-488 (green) to stain the F-actin, and 4′,6-diamidino-2-phenylindole (DAPI; blue). Non-transfected cells and cells incubated with the secondary antibody alone were included as negative controls. Images were taken under a confocal fluorescence microscope. Scale bar, 100 µm.

Plasmid transfection experiments were first performed to validate the design of the CRISPR-Cas9 construct prior to phage vector production and phage cell transduction. Therefore, the A549 human lung adenocarcinoma cells were transfected with the phage plasmid construct and immunocytochemistry was carried out 48 h post-transfection. As shown in Figure 2, cells transfected with the CRISPR-Cas9 phage plasmid expressed the Cas9 protein as compared to control non-transfected cells or cells incubated with a secondary antibody only.

**Figure 3:**
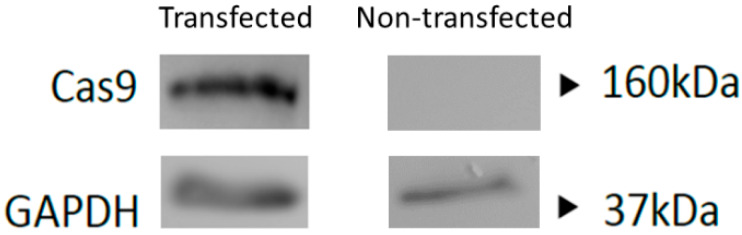
Western blot analysis of Cas9 protein (160 kDa) production in HEK293 cells at 24 h post-transfection with the phage plasmid carrying the CRISPR-Cas9 transgene cassette. Non-transfected cells were used as a negative control. GAPDH (37 kDa) was used as a loading control.

To further validate and confirm expression of Cas9 from the phage plasmid construct, we transfected HEK293 cells as a cellular model to maximize protein production and performed a Western blot analysis. The data revealed expression of the Cas9 protein at the expected 160 kDa size (Figure 3).

### 3.2. Analyzing the Impact of the CRISPR-Cas9 Transgene Cassette on Phage Vector Production and Titers Shows No Significant Alterations

**Figure 4:**
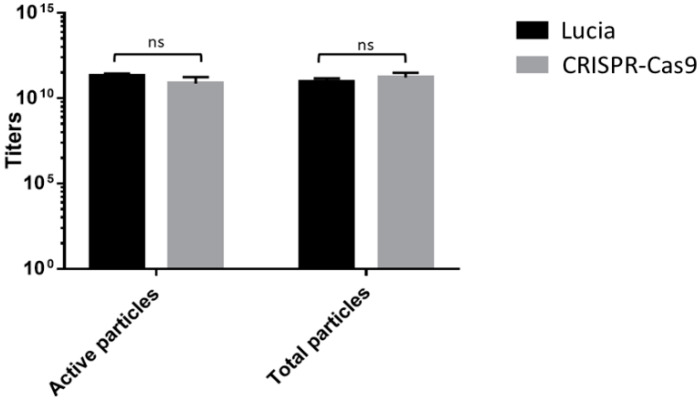
Evaluation of total phage particles as genome copies (GC/µL) by qPCR, and active phage particles (TU/µL) by agar plate counting of RGD4C phage vectors carrying either CRISPR-Cas9 or secreted luciferase (*Lucia*) transgene cassettes. A paired *t*-test showed statistically insignificant differences (ns) between the two groups of phage; *p* = 0.7883.

The large size of the CRISPR-Cas9 transgene cassette has been a challenge for vectors to accommodate and deliver [26,27,28]. We therefore sought to carry out quantitative analyses of the produced phage vector particles carrying the CRISPR-Cas9 cassette before cell transduction experiments, in order to examine possible consequences on phage vector production. We evaluated effects on the total vector genome copies and total active phage particles. Our data showed that no changes occurred in the production of active transducing phage and vector genome copies, irrespective of the genome size packaged by the phage capsid (Figure 4). Indeed, the overall active transducing particles and vector genome of phage carrying CRISPR-Cas9 were similar to that of a phage with a significantly reduced size genome containing a reporter luciferase-based transgene cassette (5274 bp) (Figure 4). These findings confirm that neither the phage vector titer nor the fraction of active functional phage were affected by cloning of the CRISPR-Cas9 transgene cassette.

### 3.3. Evaluation of Cas9 Delivery to Lung Cancer Cells by Bacteriophage Vector Carrying the CRISPR-Cas9 Cassette Shows Efficient Transduction

**Figure 5:**
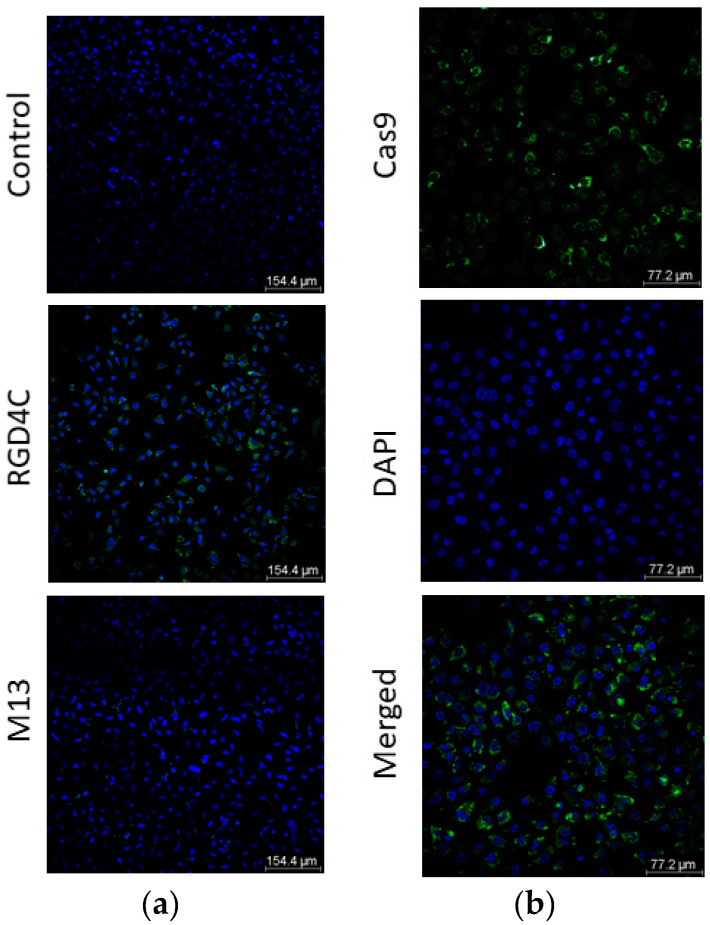
(**a**) Confocal images of immunofluorescence staining of Cas9 in A549 cells following transduction with bacteriophage-based vectors at 2,000,000 TU/cell. Cells were treated with either non-targeted (M13) or targeted (RGD4C) vectors carrying the CRISPR-Cas9 cassette. At day 6 post vector treatment, cells were stained with anti-Cas9 (green) and DAPI (blue). Cells incubated with the secondary antibody only were used as control. Scale bar, 154.4 µm; (**b**) Higher magnification image of Cas9 staining in A549 cells upon treatment with RGD4C phage vector carrying the CRISPR-Cas9. Image is separated in channels: Cas9 (green), DAPI (blue) and merged. Scale bar, 77.2 µm.

Next, we assessed the potential of RGD4C bacteriophage to target and deliver *CRISPR-Cas9* in A549 human lung cancer cells. As shown in Figure 5, we detected Cas9 expression in up to 60–70% of A549 cells at day 6 post treatment with the targeted vector. No expression was detected in cells treated with the non-targeted M13 bacteriophage-based vector carrying the CRISPR-Cas9 transgene cassette. These findings prove that delivery of CRISPR-Cas9 by the bacteriophage vector is efficient and selective, mediated by the RGD4C ligand.

### 3.4. Incorporation of a p53 gRNA in the CRISPR-Cas9 Phage Plasmid Generates Knockout Cells

**Figure 6:**
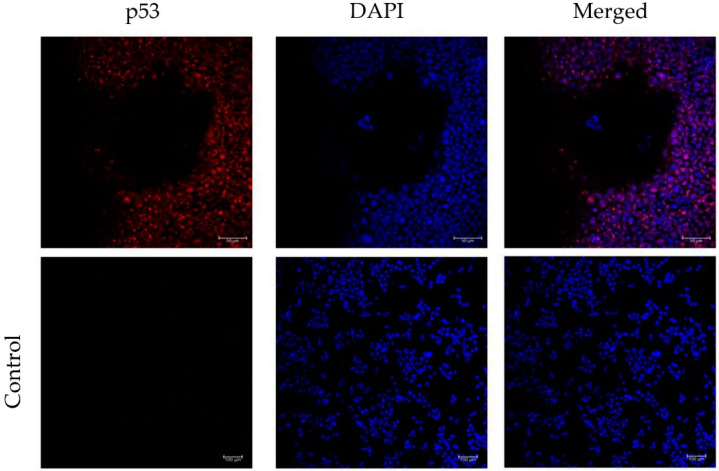
Confocal images of immunofluorescence staining of p53 localization in HEK293 cells. Incubation of cells with a secondary antibody alone was included as a negative control. Images are separated in channels: p53 (red), DAPI (blue) and merged. Scale bars are indicated.

**Figure 7:**
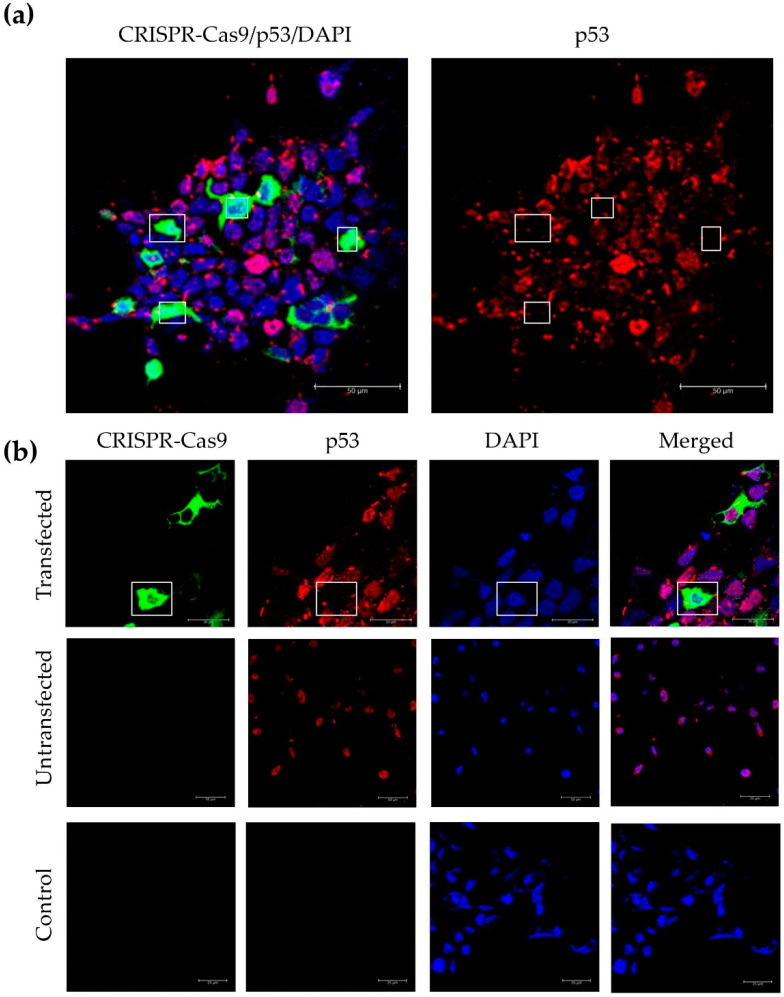
Immunofluorescence staining of Cas9 and p53. Confocal images of HEK293 cells were taken after transfection with the CRISPR-Cas9-p53gRNA phage DNA construct. (**a**) Cas9, p53 and DAPI staining were merged and compared to p53. Squares show CRISPR-Cas9-p53gRNA transfected cells, deficient in p53 protein expression. (**b**) Cas9, p53 and DAPI staining were merged and compared to Cas9, p53 and DAPI. Squares show CRISPR-Cas9-p53gRNA transfected cells, deficient in p53 expression. HEK293 cells treated with the transfection reagent PEI alone, untransfected, were also included to show the presence of p53 staining in the absence of Cas9 expression. Cells incubated with the secondary antibody only were used as a negative control. Images are separated in channels: Cas9 (green), p53 (red), DAPI (blue) and merged. Scale bars are indicated in each image.

Our initial experiments showed an accumulation of the Cas9 protein in the cytoplasm, both after vector DNA transfection and phage transduction. However, to exert its function, the CRISPR-Cas9 complex needs to reach the cell nucleus to achieve a targeted gene knockout. Consequently, we cloned a p53 gRNA in the CRISPR-Cas9 phage construct to test its effectiveness in transfected HEK293 cells. First, we carried out immunofluorescence staining using an antibody against p53, and proved expression and nuclear localization of the p53 protein (Figure 6); which was confirmed by merging with the DAPI stain commonly used as a nuclear counterstain in fluorescence microscopy. Next, importantly, we found that cells displaying nuclear expression of the CRISPR-Cas9, lacked or had negligible p53 protein expression (Figure 7a,b). In contrast, p53 expression was clearly detected in cells lacking expression of the CRISPR-Cas9 (Figure 7b).

## 4. Discussion

Here, we utilized a rational design approach to construct and characterize a genetically engineered M13 bacteriophage to deliver in vitro the CRISPR-Cas9 transgene into A549 human lung adenocarcinoma cells. This vector has proved safe in mice and pet dogs upon single and repeated intravenous administrations [11,18,23]. Interestingly, our phage-based vectors are immunogenic but they can be repeatedly administered to efficiently reach their target tissue with no off-target effects following systemic delivery [11,18,23]. The vector, called RGD4C bacteriophage, can effectively package a 10 kb vector genome. Indeed, no significant reduction was observed in either the active or total number of particles compared to the RGD4C bacteriophage carrying a luciferase transgene cassette with half the size of CRSPR-Cas9 cassette. It is noteworthy to mention that the phage DNA construct already includes a space for the gRNA target sequence, ensuring that the vector genome will not increase in size after cloning the remaining elements. In the gene therapy field, AAV vectors are among the most investigated due to their safety profile. However, in clinical trials for Haemophilia B, patients showed symptoms of inflammation and liver toxicity when large doses of the virus were injected [29]. In addition, the infection efficiency often remains low and unfortunately, due to its immunogenicity, repeated administrations are hindered by neutralizing antibodies [30]. Last, but not least, advanced strategies are required to pack and deliver large size transgenes like CRISPR-Cas9, such as delivering the gRNA by a different vector, which reduces the genome size but also compromises the efficiency of therapy.

The original aim of the study was to introduce RGD4C bacteriophage as a potential gene delivery vector in lung cancer gene editing. Altogether, our results indicate that the vector can successfully deliver the CRISPR-Cas9 transgene cassette and express the Cas9 protein in human lung adenocarcinoma cells efficiently and selectively. We also showed that the transgene cassette is functional and able to knock out p53 protein expression after adding the appropriate gRNA.

Ideally, following these initial steps, we would then include in the same vector a healthy copy of *TP53* to correct the aberrant gene. *TP53* has a major role in deciding the fate of a cell, such as cell death or cell cycle arrest. Somatic mutations in *TP53* can be decisive for the commencement of tumorigenesis, progression or malignancy. Hence, it is no doubt that p53 is a hot topic in cancer gene therapy. Challenges lie ahead on the complexity of the therapy. The basis of *TP53* gene replacement therapy consists of gene editing the *mutTP53* allele and replacing it with a healthy one. This requires gene editing tools like the CRISPR-Cas9 technology to cut out the aberrant allele and a *wt TP53* gene to replace it. Hence, viral vectors with small gene packaging size are not suitable.

Our findings, together with other features, suggest that RGD4C bacteriophage is a promising model for *TP53* gene replacement therapy. A change in the promoter choice and structural modifications of the phage capsid are already taken into consideration for an improved generation of the vector [13,23].

This project may have an interesting impact in the clinical development of new gene therapies. The RGD4C bacteriophage has shown safe systemic administration, tolerability to repeated administrations, tumour tropism and cost-effectiveness [18,19,22,31,32,33,34]. These characteristics, in addition to its high packaging size, bring a new competitive gene delivery vector to the field of CRISPR-Cas9 technology.

## 5. Conclusions

Our RGD4C bacteriophage-based vector can carry genome sizes superior to 10 kb without any significant reduction in its titers. This includes the CRISPR-Cas9 phage vector (10,183 bp) constructed in this study for a future lung cancer *TP53* gene replacement therapy. The vector can target A549 human lung adenocarcinoma cells in vitro and deliver the CRISPR-Cas9 sequence selectively with a 60–70% efficiency.
